# Markers of extracellular matrix remodeling and systemic inflammation in patients with heritable thoracic aortic diseases

**DOI:** 10.3389/fcvm.2022.1073069

**Published:** 2022-12-20

**Authors:** Bjørn Edvard Seim, Margrethe Flesvig Holt, Aleksandra Ratajska, Annika Michelsen, Monica Myklebust Ringseth, Bente Evy Halvorsen, Mona Skjelland, John-Peder Escobar Kvitting, Runar Lundblad, Kirsten Krohg-Sørensen, Liv T. N. Osnes, Pål Aukrust, Benedicte Paus, Thor Ueland

**Affiliations:** ^1^Department of Cardiothoracic Surgery, Oslo University Hospital, Rikshospitalet, Oslo, Norway; ^2^Institute of Clinical Medicine, Faculty of Medicine, University of Oslo, Oslo, Norway; ^3^Department of Cardiology, Oslo University Hospital, Rikshospitalet, Oslo, Norway; ^4^Research Institute of Internal Medicine, Oslo University Hospital, Oslo, Norway; ^5^Department of Medical Genetics, Oslo University Hospital, Oslo, Norway; ^6^Department of Neurology, Oslo University Hospital, Rikshospitalet, Oslo, Norway; ^7^Department of Immunology, Oslo University Hospital, Rikshospitalet, Oslo, Norway; ^8^Section of Clinical Immunology and Infectious Diseases, Oslo University Hospital, Rikshospitalet, Oslo, Norway; ^9^Faculty of Health Sciences, K. G. Jebsen Thrombosis Research Center, University of Tromsø – The Arctic University of Norway, Tromsø, Norway

**Keywords:** heritable thoracic aortic disease, inflammation, extracellular matrix (ECM), connective tissue disease (CTD)/collagen vascular disease (CVD), Loeys-Dietz syndrome, Marfan syndrome, aorta, familial thoracic aortic aneurysm 6

## Abstract

**Background:**

In approximately 20% of patients with thoracic aortic aneurysms or dissections a heritable thoracic aortic disease (HTAD) is suspected. Several monogenic connective tissue diseases imply high risk of aortic disease, including both non-syndromic and syndromic forms. There are some studies assessing inflammation and extracellular matrix remodeling in patients with non-hereditary aortic disease, but such studies in patients with hereditary diseases are scarce.

**Aims:**

To quantify markers of extracellular matrix (ECM) and inflammation in patients with vascular connective tissue diseases versus healthy controls.

**Methods:**

Patients with Loeys-Dietz syndrome (LDS, *n* = 12), Marfan syndrome (MFS, *n* = 11), and familial thoracic aortic aneurysm 6 (FTAA6, *n* = 9), i.e., actin alpha 2 (ACTA2) pathogenic variants, were recruited. Exome or genome sequencing was performed for genetic diagnosis. Several markers of inflammation and ECM remodeling were measured in plasma by enzyme immunoassays. Flow cytometry of T-cell subpopulations was performed on a subgroup of patients. For comparison, blood samples were drawn from 14 healthy controls.

**Results:**

(i) All groups of HTAD patients had increased levels matrix metalloproteinase-9 (MMP-9) as compared with healthy controls, also in adjusted analyses, reflecting altered ECM remodeling. (ii) LDS patients had increased levels of pentraxin 3 (PTX3), reflecting systemic inflammation. (iii) LDS patients have increased levels of soluble CD25, a marker of T-cell activation.

**Conclusion:**

Our data suggest that upregulated MMP-9, a matrix degrading enzyme, is a common feature of several subgroups of HTAD. In addition, LDS patients have increased levels of PTX3 reflecting systemic and in particular vascular inflammation.

## Introduction

Aortic diseases constitute a diverse spectrum of phenotypes ranging from aneurysms to life threatening events such as dissection. The development of aortic aneurysms is frequently asymptomatic and undiagnosed until serious complications occur (1).

Aortic aneurysms encompass complex pathophysiological features, and the underlying pathological processes are diverse and not fully elucidated. In approximately 20% of patients with thoracic aortic aneurysms or dissections a heritable thoracic aortic disease (HTAD) is suspected (2), and a monogenic cause is identified in an increasing number of these families. HTAD occur as syndromic as well as non-syndromic forms, both of which are frequently autosomal dominantly inherited. While reduced penetrance of the vascular phenotype is common in the non-syndromic forms, high penetrance is observed in some of the syndromic forms. Genetic heterogeneity of HTAD is established and includes genes affecting or interacting with transforming growth factor (TGF)-signaling [*FBN1* (3), *TGF-β2*, *TGF-β3*, *TGF-βR1*, *TGF-βR2*, *SMAD2*, and *SMAD3*), genes related to the development and function of smooth muscle cells (*MYLK*, *MYH11*, and *ACTA2*), and others, such as LOX, encoding the extracellular matrix crosslinking enzyme, lysyl oxidase. Pathogenic variants in 30 validated genes have been associated with HTAD (4).

Even though the mutated gene may point toward a causative mechanism, the pathogenesis of HTAD is not fully understood. Elucidation of the pathogenesis is pertinent to develop pharmacological treatment or prophylaxis of HTAD. Furthermore, as the disease is often asymptomatic, there is a need for non-invasive biomarkers that could monitor disease progression. There are some studies on biomarkers reflecting inflammation, extracellular matrix remodeling and thrombus formation in patients with atherosclerotic aortic aneurysms, but such studies in HTAD are scarce (5–7).

In the present study, we analyzed the plasma levels of a broad spectrum of markers reflecting inflammation, ECM remodeling and fibrogenesis and endothelial cell activation/vascular inflammation in patients with connective tissue diseases.

## Materials and methods

### Patients and healthy controls

Patients were recruited at the multidisciplinary outpatient clinic for patients with vascular connective tissue diseases at the Department of Cardiothoracic Surgery, Oslo University Hospital (OUH), a member of the European Reference Network on Rare Multisystemic Vascular Diseases (VASCERN). The patients were allocated into three groups of HTAD consisting of both syndromic [Loeys-Dietz syndrome (LDS), *n* = 12; Marfan syndrome (MFS), *n* = 11] and non-syndromic [familial thoracic aortic aneurysm 6 (FTAA6), *n* = 9] forms (Table 1). All patients were genetically characterized and had undergone exome or genome based sequencing analysis at the Department of Medical Genetics, OUH. Pathogenicity of variants were assessed by the American College of Medical Genetics and Genomics/Association for Molecular Pathology (ACMG/AMP) criteria, and all patients had a sequence variant that was assessed as pathogenic or likely pathogenic (Supplementary Table 1) (8). All MFS patients had a pathogenic variant in *FBN1*, all LDS patients had a pathogenic variant in *TGF-βR1* (*n* = 1), *TGF-βR2* (*n* = 1), *SMAD3* (*n* = 2), or *TGF-β2* (*n* = 7), and all FTAA6 patients had a pathogenic variant in *ACTA2*. For comparison, blood samples were drawn from 14 self-reported healthy subjects, with no current diseases, no chronic diseases and no regular medications. The study was approved by the Regional Committee for Medical and Health Research Ethics in South East Norway (REC no. 2018/732). Written informed consent was obtained from all participants.

**TABLE 1:** Values are presented as number (%) or median [25–75 percentile].

	Healthy controls *n* = 14	FTAA6 *n* = 9	LDS *n* = 12	MFS *n* = 11
Clinical characteristics				
Age, years	54 [52–55]	50 [31.5–63]	39.5 [28.8–54.7]*	35 [29–48]*
BMI, kg/m^2^	25 [22–26]	24 [23–25]	26.5 [23–35]	22 [19–27]
Gender, men, *n*	6 (43)	4 (44)	5 (36)	5 (45)
Hypertension, *n*	0 (0)	3 (33)*	3 (25)*	0 (0)
Hyperlipidemia, *n*	0 (0)	1 (11)	1 (8)	2 (18)
Atrial fibrillation, *n*	0 (0)	0 (0)	1 (8)	1 (9)
Previous aortic surgery, *n*	0 (0)	1 (11)	3 (25)*	5 (45)*
Acute dissection, TAA repair, AAA repair, *n*	0/0/0	1/0/0	0/3/0	0/4/1
**Biochemistry**				
WBC (× 10^9^/L)	5.4 [4.6–7.3]	6.7 [5.9–6.9]	7.2 [5.5–8.3]	7.4 [6.0–9.1]*
eGFR, ml/min/1.73^2^	98 [85–101]	105 [87–108]	108 [97–113]*	108 [91–120]
**Medication, *n***				
Antiplatelet therapy	0 (0)	1 (11)	3 (25)*	0 (0)
ACE inhibitors/ARBs	0 (0)	3 (33)*	4 (33)*	1 (9)
Beta-blockers	0 (0)	2 (22)	4 (33)*	5 (45)*
Statins	0 (0)	1 (11)	1 (8)	2 (18)
NSAIDS	0 (0)	0 (0)	1 (8)	0 (0)

*p < 0.05 vs. healthy controls. LDS, Loeys-Dietz syndrome; MFS, Marfan syndrome; FTAA6, familial thoracic aortic aneurysm 6; HC, healthy controls; BMI, body mass index; TAA, thoracic aortic aneurysm; AAA, abdominal aortic aneurysm; WBC, white blood cell count; eGFR, estimated glomerular filtration rate; ACE, angiotensin-converting enzyme; ARB, angiotensin receptor blocker.

### Selection of markers

We selected the markers we analyzed on the basis of two criteria. Firstly, we chose markers involved in pathophysiological processes related to HTAD, i.e., extracellular vascular remodeling/fibrogenesis, general and vascular inflammation, immune/T-cell activation. Then, we did a search in literature in relation to MFS, LDS, and ACTA2 variant-positive HTAD, to determine whether or not the selected markers represent originality in these disorders.

### Blood sampling protocol and biochemical analyses

Plasma samples collected by venipuncture in sterile EDTA tubes were placed on melting ice, centrifuged within 30 min at 2,000*g* for 20 min to obtain platelet-poor plasma and stored at −80°C. All samples were thawed only once. Detailed information about the biomarkers is presented in Table 2. Plasma levels of vWF and fibronectin were measured by enzyme immunoassays (EIA) using antibodies from DAKO (Agilent, Santa Clara, CA, USA), all other markers, listed in Table 2, were measured using antibodies from R&D Systems (Minneapolis, MN, USA), in a 384-format using a combination of a SELMA pipetting robot (Analytic Jena AG, Jena, Germany) and a BioTek dispenser/washer (BioTek Instruments, Winooski, VT, USA). Intra- and inter-assay CVs were <10% for all assays.

**TABLE 2:** Markers of extracellular matrix remodeling, vascular inflammation, and immune activation.

Biomarkers	Description and functions
**Markers of ECM remodeling/fibrosis**	
TGF-β1	TGF-β1 is expressed in several tissues. It has anti-inflammatory functions, but also potential profibrotic effects. TGF-β1’s functions are inseparably linked to the function of regulatory T-cells (24).
TGF-βR2	A single transmembrane protein with a cytoplasmic serine/threonine kinase domain. Loss of function mutations in the gene encoding TGF-βR2 cause LDS (25).
TGF-β3	Cytokine believed to play an important role in fibrosis, formation of ECM and cellular adhesion (26).
Matrix metalloproteinase-9 (MMP-9)	An important function of MMP-9 is to degrade ECM-components. MMP-9 is upregulated in several inflammatory diseases (23).
Growth differentiation factor 15 (GDF-15)	Member of the TGF-β superfamily involved in ECM remodeling and inflammation, and high levels are reported in heart diseases, inflammatory disorders, and cancer (27).
Cystatin B (CysB)	Intracellular cysteine protease inhibitor and expression is increased upon cellular stress and activation of macrophages. Central player in inflammation and fibrogenesis (28).
Tissue inhibitor of metalloproteinases 2 (TIMP-2)	Capable of both inhibiting and activating MMPs, and is the only TIMP which interacts with a cell-membrane bound MMP (29).
Fibronectin (FN1)	FN1 is an extra cellular matrix protein important in cell adhesion, migration, growth, and differentiation (30).
**Vascular inflammation**	
Osteoprotegerin (OPG)	OPG is a cytokine receptor and member of the TNF receptor-superfamily, expressed in several tissues and is linked to vascular inflammation (31).
CXC chemokine ligand 16 (CXCL16)	CXCL16 promotes recruitment of leukocytes into the vascular bed, contributing to vascular inflammation (32).
Von Willebrand factor (vWF)	The major cellular source of vWF is an activated endothelium and released vWF promote platelet activation (33).
Vascular cell adhesion molecule 1 (VCAM-1)	VCAM-1 is an adhesion molecule, induced by various inflammatory mediators, for lymphocytes migrating from blood to tissue. Through the VCAM-1 pathway ROS-production is stimulated, which subsequently activates MMPs (34).
Inflammation and immune activation	
C-reactive protein (CRP)	An established and reliable marker of upstream inflammation (35).
Pentraxin 3 (PTX3)	A soluble pattern recognition receptor that, in contrast to CRP, is produced at the site of inflammation including vascular inflammation (36).
sCD14	Soluble (s) CD14 is a general marker of monocyte activation (37).
sCD163	Soluble CD163 is another marker of macrophage activation thought to reflect a pro-resolving monocyte subset (37).
Soluble T-cell immunoglobulin mucin domain-3 (sTIM-3)	sTIM-3 is proposed to serve as a biomarker of immune and in particular T-cell exhaustion (38).
sCD25	Also named sIL-2 receptor α, is an established marker of T-cell activation (39).
Myeloperoxidase (MPO)	Is an enzyme peroxidase mainly released by neutrophils, providing defense against pathogens. Active MPO is present in atherosclerotic plaques with higher levels if MPO-expressing macrophages in more advanced plaques (40).

ECM, extracellular matrix; TGF, transforming growth factor; TNF, tumor necrosis factor.

### Flow cytometry of T cells and T-cell subpopulations

Flow cytometry of absolute counts for T cells were analyzed in Trucount tubes (BD, Franklin Lakes, NJ, USA) on a FacsCanto II instrument and analyzed in BD FACSCanto™ Clinical Software according to the instructions provided by the manufacturer (BD). Instrument settings were standardized as recommended by the manufacturer with daily quality run with CS&T Beads (BD) and 7-color Setup Beads (BD) ensuring high reproducibility. The laboratory follows standard operation procedure and also has ISO (International Standard Organization) certification. Further sub-classification of T cells was performed on a Gallios Flow cytometer (Beckman Coulter, San Diego, CA, USA) as previously described (9). Reference values (5–95 percentile) for absolute numbers of T and NK-cells, and T-subpopulations were established on samples from healthy blood donors (*n* = 65).

### Statistics

**FIGURE 1:**
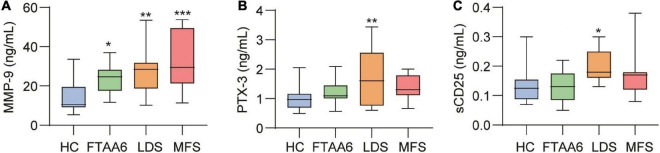
Results from EIA analyses of **(A)** MMP-9, **(B)** PTX3, and **(C)** sCD25. *p < 0.05, **p < 0.01, ***p < 0.001 vs. healthy controls (HC). MMP-9, matrix metalloproteinase-9; PTX3, pentraxin 3; LDS, Loeys-Dietz syndrome; MFS, Marfan syndrome; FTAA6, familial thoracic aortic aneurysm 6.

Demographics between the different diagnostic groups versus healthy controls were compared using Kruskal–Wallis *a priori*, and if significant, differences between groups were compared with the Mann–Whitney U-test. Categorical variables were compared using a chi square test. The distribution of TGF-β1, TGF-βR2, TGF-β3, sCD25, sTIM-3, cystatin B, sCD14, matrix metalloproteinase-9 (MMP-9), and CRP was skewed and log10 transformed. Markers were compared between groups by multivariate general linear model with age and BMI as covariates. *Post hoc* analyses are presented for markers with *p* < 0.1 for diagnostic group. Markers are in tables presented as estimated marginal means with 95% confidence intervals in Table 3 and as Tukey box plots in Figure 1. We did not correct for multiple testing. A two-sided *p* < 0.05 was considered significant.

**TABLE 3:** Results from enzyme immunoassays (EIA) analyses of the main findings.

	Healthy controls *n* = 14	FTAAD *n* = 9	LDS *n* = 12	MFS *n* = 11	*p*
**Markers of ECM remodeling/fibrosis**					
TGF-β1, ng/ml	2.7 (1.8–4.0)	1.7 (1.1–2.6)	2.1 (1.4–3.3)	2.2(1.5−−3.5)	0.47
TGF-βR2, ng/ml	0.38 (0.30–0.49)	0.35 (0.27–0.47)	0.45 (0.35–0.58)	0.43(0.33−−0.56)	0.61
TGF-β3, ng/ml	184 (81–417)	80 (32–204)	74 (31–177)	179(74−−430)	0.26
MMP-9, ng/ml	12 (9–17)	22 (16–32)*	27 (19–37)**	29(21−−40)***	0.004
GDF-15, ng/ml	0.25 (0.21–0.30)	0.27 (0.22–0.33)	0.32 (0.26–0.38)	0.28(0.23−−0.35)	0.45
Cystatin B, ng/ml	1.9 (1.5–2.5)	2.6 (2–3.5)	3.1 (2.3–4)	2.8(2.1−−3.7)	0.12
TIMP-2, ng/ml	96 (85–107)	90 (78–102)	110 (99–122)	107(96−−119)	0.085
FN1, arb. unit	0.27 (0.22–0.31)	0.24 (0.19–0.3)	0.26 (0.21–0.31)	0.29(0.24−−0.34)	0.55
**Vascular inflammation**					
OPG, ng/ml	0.43 (0.37–0.49)	0.43 (0.36–0.5)	0.45 (0.38–0.51)	0.5(0.43−−0.57)	0.40
CXCL16, ng/ml	1.14 (1.04–1.24)	1 (0.89–1.12)	1.11 (1.01–1.22)	1.23(1.12−−1.34)	0.047
vWF, % of ref.	160 (80–240)	181 (89–272)	169 (84–255)	221(134−−307)	0.76
VCAM-1, ng/ml	324 (257–392)	339 (262–415)	325 (254–396)	361(289−−433)	0.86
**Inflammation and immune activation**					
CRP, μg/ml	0.27 (0.16–0.45)	0.34 (0.19–0.62)	0.74 (0.42–1.29)*	0.49(0.28−−0.86)	0.092
PTX3, ng/ml	0.96 (0.62–1.31)	1.21 (0.81–1.6)	1.69 (1.33–2.06)**	1.47(1.1−−1.84)	0.054
sCD14, ng/ml	1.9 (1.5–2.4)	1.7 (1.3–2.2)	2.4 (1.9–3)	2.3(1.8−−3)	0.21
sCD163, ng/ml	169 (117–220)	190 (131–249)	188 (133–243)	206(151−−262)	0.815
sTIM-3, ng/ml	3.1 (2.4–4)	3.1 (2.3–4.1)	3.8 (2.9–4.9)	3.6(2.8−−4.7)	0.66
sCD25, ng/ml	0.13 (0.09–0.17)	0.13 (0.09–0.18)	0.2 (0.16–0.24)*	0.18(0.14−−0.22)	0.084
MPO, ng/ml	13 (9–16)	17 (13–22)	17 (13–21)	18(14−−22)	0.19

Plasma levels of several markers are increased compared to healthy controls. *p < 0.05, **p < 0.01, ***p < 0.001 vs. HC. LDS, Loeys-Dietz syndrome; MFS, Marfan syndrome; FTAA6, familial thoracic aortic aneurysm 6; TGF-β1, transforming growth factor beta 1; TGF-βR2, transforming growth factor beta receptor 2; TGF-β3, transforming growth factor beta 3; MMP-9, matrix metalloproteinase 9; GDF-15, growth/differentiation factor 15; TIMP-2, tissue inhibitor of metalloproteinases 2; FN1, fibronectin; OPG, osteoprotegerin; CXCL16, CXC chemokine ligand 16; vWF, Von Willebrand factor; VCAM-1, vascular cell adhesion molecule 1; CRP, C-reactive protein; PTX3, pentraxin 3; sCD14, soluble CD14; sCD163, soluble sCD163; sTIM-3, soluble T-cell immunoglobulin mucin domain-3; sCD25; soluble CD25; MPO, myeloperoxidase.

## Results

The demographic characteristics of the three HTAD groups, i.e., LDS, MFS, and FTAA6 as well as healthy controls are shown in Table 1. Prior to inclusion, one of the FTAA6, three of the LDS, and five MFS patients had undergone aortic surgery. All, except from one MFS patient had undergone surgery on the thoracic aorta, with valve-sparing aortic root replacement being the most frequent. One patient had been operated due to a type A aortic dissection. The patients had no known cancers and none had inflammatory diseases that were unrelated to their HTAD. The LDS and MFS group was significantly younger than controls. The results from EIA analyses of the different groups are shown in Table 3 and Figure 1.

### Markers of extracellular matrix remodeling

As shown in Figure 1A, all patient groups had significantly elevated levels of MMP-9 compared to healthy controls, in age-adjusted analysis, with the highest levels in MFS patients (Table 3).

### Markers of general and vascular inflammation

As outlined in Table 3, patients with LDS had significantly higher levels of CRP and pentraxin 3 (PTX3) (Figure 1B) as compared with healthy controls. As for the other inflammatory markers including those that reflect activation of monocytes/macrophages (sCD14 and sCD163) and neutrophils (MPO), the HTAD patients had no increase as compared with healthy controls (Table 3).

### Markers of T-cell activation

LDS patients had higher levels of sCD25, reflecting T-cell activation, compared to healthy controls (Table 3 and Figure 1C). However, there were no difference in sTIM-3, reflecting T-cell exhaustion. As shown in Supplementary Table 2, flow cytometry of T-cell subpopulations revealed some findings of potential interest. While we observed no significant differences between the three diseases, in the patient group as a whole, 43% of the patients had an increased proportion of CD4^+^ memory T cells. Sixty two percent had an increased proportion of CD8^+^ early effector/memory T cells, with a similar pattern in LDS, MFS and FTAA6 (Supplementary Table 2).

### MMP-9 and PTX3 levels in phenotype-negative HTAD patients

Five of the patients (two FTAA6, two LDS, and one MFS) had no clinical or radiological abnormalities and were followed because of a pathogenic variant detected in symptomatic relatives. As shown in Supplementary Figure 1, whereas these five patients had numerically higher levels of MMP-9 and PTX3 than healthy controls, and for PTX3 with similar mean levels as in the symptomatic patients, the differences did not reach statistical significance, most probably reflecting the low number of phenotype-negative patients.

When comparing proportions in T-cell population between symptomatic and phenotype-negative patients we found that patients with symptoms had more late CD8^+^ effector/memory cells (*p* = 0.014) and less regulatory T cells (*p* = 0.006) (Supplementary Table 3), suggesting a higher degree of T-cell activation in the symptomatic patients.

## Discussion

Although genetic causes were established in all patients included in the present study (MFS: *FBN1*, LDS: pathogenic variants in several TGF-β pathway genes, FTAA6: *ACTA2)*, this may not necessarily reflect all pathogenic mechanisms that are activated in these patients. The major finding in the present study was increased levels of MMP-9, a major regulator of ECM remodeling, in all sub-groups of HTAD patients. In addition, we found raised levels of PTX3, a marker of systemic inflammation in LDS patients. Both MMP-9 and PTX3 could be of relevance for the pathogenesis of aortic aneurysms, potentially through interacting mechanisms.

Pathological ECM remodeling is involved in aneurysm development including those in HTAD of both syndromic and non-syndromic forms (10). In the present study we found that all patient groups had significantly elevated MMP-9 levels as compared to healthy controls, also after adjustment for age, gender and BMI, with particularly high levels in patients with MFS and LDS. MMP-9 is a prototypical matrix-degrading enzyme that could be activated by inflammation and could in itself promote inflammation, representing a pathogenic loop in ECM remodeling (11, 12). Such pathogenic mechanisms could potentially be present in HTAD as well. Elevated MMP-9 levels have been reported in MFS (13) whereas the data in LDS are scarce and normal levels have been reported in a small cohort of children (14). However, this is, to the best of our knowledge, the first report on elevated MMP-9 levels in FTAA6. There are some data on increasing MMP levels with age (15), and we cannot exclude that such mechanisms could have contributed to different results in publications on MMP levels. Nonetheless, in the present study, when comparing patients and controls, results were adjusted for age.

Inflammation has been implicated in the pathogenesis of HTAD (16) and in the present study we show that LDS patients had increased levels of CRP and in particular of PTX3 as compared with healthy controls. In contrast to CRP, PTX3 is produced at the site of inflammation, and high levels have been found in various forms of vasculitis and in atherosclerotic abdominal aorta (17, 18). Herein, we report that LDS patients also have elevated PTX3 levels. PTX3 is expressed in various organs, and with relevance to HTAD, it is up-regulated in endothelial cells during inflammation (19). One could speculate that the increased levels in LDS could reflect enhanced inflammation in the thoracic aorta potentially contributing to the pathogenesis of this manifestation, but to the best of our knowledge, data on PTX3 expression in vascular tissue from HTAD patients are lacking. However, a recent study by Lei *et al.* showed that PTX3 was significantly overexpressed in ruptured as compared with stable abdominal aortic aneurysms (20). Although the study was not performed in thoracic aneurysms, it illustrates a potential involvement of PTX3 in the pathogenesis of human aneurysm development.

Enhanced expression of MMP-9 and PTX3 has been reported in various inflammatory and autoimmune disorders as well as in malignancies and end-stage kidney disease (21, 22). PTX3 is not only a marker but also a potential mediator in these disorders, and notably, PTX3 seems to be involved not only in inflammation as being part of the innate immunity, but also in ECM remodeling at least partly by the induction of MMPs including MMP-9 (22). It could therefore be hypothesized that the bidirectional interaction between MMP-9 and PTX3 could play a role in the pathogenesis of subgroups of HTAD patients such as LDS.

Data on the role of T cells in MFS and LDS are scarce or lacking. Interestingly, treatment with methotrexate have been shown to prevent aortic dilation in a murine model of MFS potentially involving modulation of T-cell activity (23). Herein we show that patients with LDS had higher plasma levels of sCD25 suggesting enhanced T-cell activation. Furthermore, HTAD patients had increased proportion of CD4^+^ memory T cells and CD8^+^ early effector/memory T cells that may suggest persistent antigen exposure and T-cell activation. However, at present the role of T cells in the pathogenesis of HTAD remains unclear.

Our data may suggest that the LDS patients represent a particular inflammatory subgroup within the spectrum of HTAD. In addition to raised levels of MMP-9 they also had raised levels the pentraxins CRP and PTX3 as well as sCD25, a marker of T-cell activation. The reason for this pattern is at present not clear, but could be related to their pathogenic variants in TGFRs that in addition to mediating tissue repair also have some anti-inflammatory potentials (24).

The present study has some important limitations. The cohort constitute few patient and our results must be interpreted with caution. Due to the limited population size and the explorative nature on the study we did not correct for multiple testing. However, clearly our finding of elevated MMP-9 levels in all diagnostic groups is the most robust. Furthermore, the patients were not followed prospectively over time with serial assessment of soluble biomarkers. Moreover, association does not necessarily mean any causal relationship. Finally, the lack of data on the expression of these markers in tissue samples from these patient groups also limit the importance of our findings.

Our data show elevated plasma levels of MMP-9, a mediator involved in both inflammation and ECM remodeling, across all sub-groups of HTAD patients when compared to healthy controls. In addition, LDS patients also had elevated levels of PTX3 that together with MMP-9 might represent two interacting arms in the pathogenesis of this subgroup of HTAD patients. However, larger prospective studies, as well as mechanistic studies are needed to clarify if these pathways could represent non-invasive prognostic biomarkers or therapeutic targets in these patients.

## Data availability statement

The Regional Committees for Medical Research Ethics South East Norway (REK South East) approved the conduction of the study. A condition for approval was that privacy concerns were respected and that data were not made publicly available. However, excerpts of de-identified data relevant to the study can be made available upon reasonable request. Requests to access the datasets should be directed to MH, mafhol@ous-hf.no.

## Ethics statement

The studies involving human participants were reviewed and approved by the Regional Committees for Medical Research Ethics South East Norway (REK South East). The patients/participants provided their written informed consent to participate in this study.

## Author contributions

BS, KK-S, MH, PA, TU, and BP conceived and designed the research. BS, MR, J-PK, RL, BP, AR, and KK-S established the biobank and database, included the patients, and collected the material. MS included the healthy controls, collected the material, and registered their clinical data. BP, BH, and AM contributed to reagents and material. AM and TU prepared the samples and performed EIA experiments. LO performed and interpreted flow cytometry data. BP and AR oversaw exome/genome based sequencing analysis and assessed pathogenicity of the observed variants according to the ACMG/AMP criteria. MH and TU performed the statistical analysis. TU, PA, and BP interpreted the results. BS, MH, PA, BP, and TU wrote the first draft of the manuscript. All authors contributed to manuscript revision, read, and approved the submitted version.

## Supplementary material

Supplementary Figure 1 Plasma levels of **(A)** MMP-9, and **(B)** PTX in symptomatic and phenotype negative patients. **p* < 0.05, ****p* < 0.001 *vs*. healthy controls.
