# Supplementary figures and images for: Markers of extracellular matrix remodeling and systemic inflammation in patients with heritable thoracic aortic diseases
Source: Front Cardiovasc Med. 2022 Dec 20;9:1073069. doi: 10.3389/fcvm.2022.1073069 (PMC9808784; doi:10.3389/fcvm.2022.1073069)

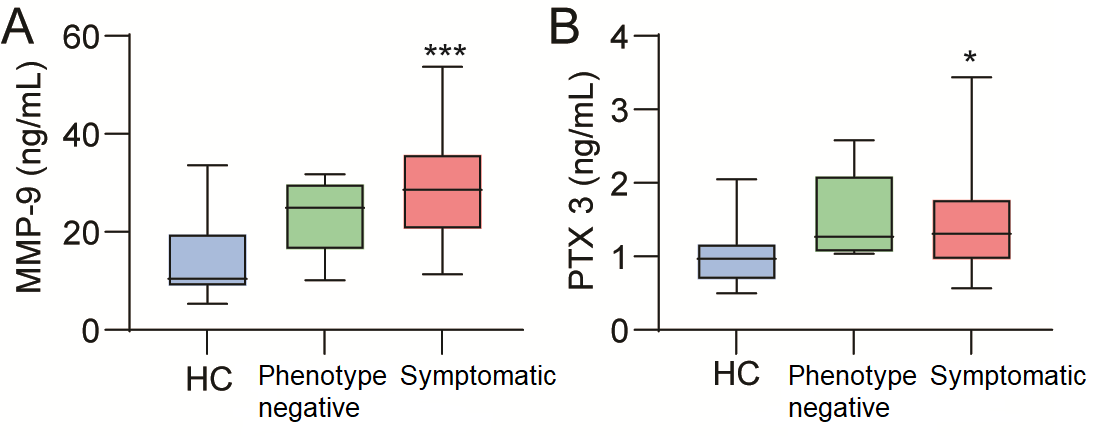

Supplement: Supplementary Figure 1 — Plasma levels of (A) MMP-9, and (B) PTX in symptomatic and phenotype negative patients. *p < 0.05, ***p < 0.001 vs. healthy controls. [file Image_1.TIF]
