# Supplementary material for: Markers of extracellular matrix remodeling and systemic inflammation in patients with heritable thoracic aortic diseases
Source: Front Cardiovasc Med. 2022 Dec 20;9:1073069. doi: 10.3389/fcvm.2022.1073069 (PMC9808784; doi:10.3389/fcvm.2022.1073069)
Supplement: Supplementary file 2 [file Table_1.docx]

**Matrix Markers in Heritable TAD**

**Supplementary Table 1**

| **Gene** | **Reference sequence** | **Nucleotide change** | **Predicted amino acid change** | **ACMG/AMP variant class** | **ACMG/AMP Evidence criteria** |
| --- | --- | --- | --- | --- | --- |
| *FBN1* | NM_00138.4 | c.653del | p.Gly218Alafs112 | 5 | PVS1- very strong, PM6- moderate |
|  |  | c.718C>T | p.Arg240Cys | 5 | PS4- strong, PM1-moderate, PM2-supporting, PP1-moderate |
|  |  | c.1511G>A | p.Cys504Tyr | 5 | PM6-strong, , PM1-strong, PS4-moderate, PM5- moderate, PM2-supporting, PP3- supporting |
|  |  | c.1539-1G>C |  | 4 | PVS1-strong, PM2- supporting, PP4- supporting, PS1- supporting |
|  |  | c.1794C>G | p.Cys598Trp | 4 | PM1- strong, PM2- supporting, PP3- supporting |
|  |  | c.3463+1G>C |  | 4 | PVS1- strong, PM2- supporting, PP4- supporting, PS4- supporting |
|  |  | c.6458G>T | p.Cys2153Phe | 4 | PM1- strong, PM2- supporting, PP3- supporting |
|  |  | c.6684T>G | p.Tyr2228 | 4 | PVS1- very strong, PM2- supporting |
|  |  | c.7708G>A | p.Glu2570Lys | 5 | PM1- strong, PS4- strong, PM2- supporting PP3- supporting, |
|  |  | c.7816_7817del | p.Val2606 | 4 | PVS1- very strong, PM2- supporting |
| *ACTA2* | NM_001613.2 | c.773G>A | p.Arg258His | 4 | PM1- moderate, PM5- moderate, PM2- supporting, PS3- supporting, PS4- supporting |
| *ACTA2* | NM_001141945.2 | c.977C>A | p.Thr326Asn | 4 | PS4- strong, PM1- moderate, PP3- supporting |
| *TGFB2* | NM_003238.3 | c.346+1G>C |  | 4 | PVS1- strong, PM2- supporting, PP5- supporting |
|  |  | c.391C>T | p.Arg131 | 5 | PVS1-very strong, PM2- supporting, PS4- supporting |
| *SMAD3* | NM_005902.3 | c.269G>A | p.Arg90His | 4 | PP1- moderate, PS4- moderate, PM2- supporting, PM5- supporting |
|  |  | c.1137_1140del | p.Trp380Glufs9 | 4 | PVS1- strong, PM1- supporting, PM2-supporting |
| *TGFBR2* | NM_003242.6 | c.1273A>G | p.Met425Val | 4 | PS4- strong, PM1- supporting, PM5- supporting, PP3- supporting, PM2- supporting |
| *TGFBR1* | NM_004612.2 | c.700T>C | p.Phe234Leu | 4 | PS4- moderate, PM1- supporting, PM2- supporting, PP3- supporting |

**Supplementary table 1.** Genetic variants in study participants and variant interpretation according to current guidelines from Richards et. al (8). The laboratory classifies variants fulfilling one strong and one supporting evidence criteria as likely pathogenic. Abbreviations ACMG, American College of Medical Genetics; AMP, Association for Molecular Pathology

**Matrix Markers in Heritable TAD**

**Supplementary Table 2**

|  |  |  | **All**  **(n = 21)** |  | **FTAAD6**  **(n = 7)** |  | **LD**  **(n = 7)** |  | **MFS**  **(n = 7)** |  |  |
| --- | --- | --- | --- | --- | --- | --- | --- | --- | --- | --- | --- |
| Cell type (T-cells) | Unit | Normal range | Mean±SD | % L/N/H | Mean±SD | % L/N/H | Mean±SD | % L/N/H | Mean±SD | % L/N/H | p-value |
| CD3+ | Cells/ μL | 900 - 4500 | 1525±379 | 5/95/0 | 1343±281 | 0/100/0 | 1597±499 | 14/86/0 | 1651±329 | 0/100/0 | 0.79 |
| CD4+ | Cells/ μL | 500 - 2400 | 946±225 | 0/100/0 | 868±210 | 0/100/0 | 957±206 | 0/100/0 | 1011±289 | 0/100/0 | 0.87 |
| CD4 + | % of total CD3+ | 22 - 66 | 49±8 | 0/95/5 | 48±7 | 0/100/0 | 46±19 | 29/71/0 | 48±17 | 17/83/0 | 0.45 |
| CD8+ | Cells/ μL | 300 - 1600 | 566±205 | 5/95/0 | 471±132 | 0/100/0 | 627±302 | 14/86/0 | 584±126 | 0/100/0 | 0.70 |
| CD8 + | % of total CD3+ | 9 - 49 | 29±10 | 0/95/5 | 26±7 | 0/100/0 | 33±13 | 0/86/14 | 29±10 | 0/100/0 | 0.43 |
| Follicular CD4+ | % of CD4+ | 6 – 72 | 8.8±3 | 10/91/0 | 9.6±4.3 | 14/86/0 | 8.8±2.8 | 14/86/0 | 8.1±1.2 | 0/100/0 | 0.47 |
| CD4+ naïve | % of CD4+ | 52 - 92 | 57±13 | 29/71/0 | 53±12 | 43/57/0 | 63.3±16.0 | 14/86/0 | 54.6±11.6 | 17/83/0 | 0.44 |
| CD4+ memory | % of CD4+ | 15 - 56 | 58±14 | 0/57/43 | 63±17 | 0/57/43 | 55±13 | 0/57/42 | 56±11 | 0/67/33 | 0.93 |
| Recent Thymic Emigrants | % of CD4+ | 37 - 100 | 48±17 | 24/76/0 | 49±18 | 29/71/0 | 46±19 | 29/71/0 | 48±17 | 17/83/0 | 0.34 |
| Regulatory | % of CD4+ | 3 - 17 | 6.2±1.8 | 0/100/0 | 6.9±2.1 | 0/100/0 | 5.7±1.4 | 0/100/0 | 5.8±2.2 | 0/100/0 | 0.55 |
| Double-negative | % of t-cells | 1 – 7 | 0.55±0.3 | 91/10/0 | 0.44±0.22 | 100/0/0 | 0.57±0.41 | 71/29/0 | 0.70±0.23 | 100/0/0 | 0.64 |
| CD8+ naïve | % of CD8+ | 54 – 88.4 | 58±13 | 0/100/0 | 58±12 | 0/100/0 | 61±17 | 0/100/0 | 59±9 | 0/100/0 | 0.84 |
| Early CD8+ Effector/memory | % of CD8+ | 1 – 9 | 10.1±4.4 | 0/38/62 | 10.9±4.5 | 0/43/57 | 10.5±5.8 | 0/42/57 | 9.7±2.0 | 0/17/83 | 0.49 |
| Late CD8+  Effector/memory | % of CD 8+ | 10 - 55 | 27±13 | 10/91/0 | 28±13 | 14/86/0 | 24±16 | 14/86/0 | 27±8 | 0/100/0 | 0.79 |

**Supplementary table 2.** Flow Cytometry % L/N/H; percentage low/normal/high levels *vs.* normal range. Abbreviations: LDS, Loeys-Dietz syndrome; MFS, Marfan syndrome; FTAA6, familial thoracic aortic aneurysm 6;

**Supplementary Table 3**

|  |  |  | **Symptomatic**  **(n = 21)** |  | **Phenotype-negative**  **(n = 5)** |  |  |
| --- | --- | --- | --- | --- | --- | --- | --- |
| Cell type (T-cells) | Unit | Normal range | Mean±SD | % L/N/H | Mean±SD | % L/N/H |  |
| CD3+ | Cells/ μL | 900 - 4500 | 1509±402 | 7/93/0 | 1569±384 | 0/100/0 | 0.89 |
| CD4+ | Cells/ μL | 500 - 2400 | 953±253 | 0/100/0 | 910±162 | 0/100/0 | 0.49 |
| CD4 + | % of total CD3+ | 22 - 66 | 49±9 | 0/93/7 | 48±5 | 0/100/0 | 0.98 |
| CD8+ | Cells/ μL | 300 - 1600 | 533±187 | 7/93/0 | 638±271 | 0/100/0 | 0.49 |
| CD8+ | % of total CD3+ | 9 - 49 | 28±9 | 0/100/0 | 34±13 | 0/80/20 | 0.23 |
| Follicular CD4+ | % of CD4+ | 6 – 72 | 8.2±2.2 | 13/87/0 | 11.0±4.3 | 0/100/0 | 0.086 |
| CD4+ naïve | % of CD4+ | 52 - 92 | 58±14 | 27/73/0 | 53±12 | 20/80/0 | 0.38 |
| CD4+ memory | % of CD4+ | 15 - 56 | 59±14 | 0/53/47 | 54±14 | 0/80/20 | 0.73 |
|  |  |  |  |  |  |  |  |
| Recent Thymic Emigrants | % of CD4+ | 37 - 100 | 44±19 | 33/67/0 | 59±7 | 0/100/0 | 0.16 |
| Regulatory | % of CD4+ | 3 - 17 | 5.6±1.6 | 0/100/0 | 7.9±1.8 | 0/100/0 | 0.006 |
| Double-negative | % of t-cells | 1 – 7 | 0.60±0.34 | 87/13/0 | 0.46±0.15 | 100/0/0 | 0.19 |
| CD8+ naïve | % of CD8+ | 54 – 88.4 | 56±12 | 0/100/0 | 69±8 | 0/100/0 | 0.070 |
| Early CD8+ Effector/memory | % of CD8+ | 1 – 9 | 9.5±3.9 | 0/40/60 | 13.1±4.5 | 0/20/80 | 0.16 |
| Late CD8+  Effector/memory | % of CD 8+ | 10 - 55 | 30±11 | 7/93/0 | 14±7 | 20/80/0 | 0.014 |
|  |  |  |  |  |  |  |  |

**Supplementary table 3.** Flow Cytometry % L/N/H; percentage low/normal/high levels *vs.* normal range. Symptomatic patients vs. phenotype-negative patients.
